# A Comparative Analysis of Disaster Risk, Vulnerability and Resilience Composite Indicators

**DOI:** 10.1371/currents.dis.453df025e34b682e9737f95070f9b970

**Published:** 2016-03-14

**Authors:** Benjamin Beccari

## Abstract

Introduction: In the past decade significant attention has been given to the development of tools that attempt to measure the vulnerability, risk or resilience of communities to disasters. Particular attention has been given to the development of composite indices to quantify these concepts mirroring their deployment in other fields such as sustainable development. Whilst some authors have published reviews of disaster vulnerability, risk and resilience composite indicator methodologies, these have been of a limited nature. This paper seeks to dramatically expand these efforts by analysing 106 composite indicator methodologies to understand the breadth and depth of practice.

Methods: An extensive search of the academic and grey literature was undertaken for composite indicator and scorecard methodologies that addressed multiple/all hazards; included social and economic aspects of risk, vulnerability or resilience; were sub-national in scope; explained the method and variables used; focussed on the present-day; and, had been tested or implemented. Information on the index construction, geographic areas of application, variables used and other relevant data was collected and analysed.

Results: Substantial variety in construction practices of composite indicators of risk, vulnerability and resilience were found. Five key approaches were identified in the literature, with the use of hierarchical or deductive indices being the most common. Typically variables were chosen by experts, came from existing statistical datasets and were combined by simple addition with equal weights. A minimum of 2 variables and a maximum of 235 were used, although approximately two thirds of methodologies used less than 40 variables. The 106 methodologies used 2298 unique variables, the most frequently used being common statistical variables such as population density and unemployment rate. Classification of variables found that on average 34% of the variables used in each methodology related to the social environment, 25% to the disaster environment, 20% to the economic environment, 13% to the built environment, 6% to the natural environment and 3% were other indices. However variables specifically measuring action to mitigate or prepare for disasters only comprised 12%, on average, of the total number of variables in each index. Only 19% of methodologies employed any sensitivity or uncertainty analysis and in only a single case was this comprehensive.

Discussion: A number of potential limitations of the present state of practice and how these might impact on decision makers are discussed. In particular the limited deployment of sensitivity and uncertainty analysis and the low use of direct measures of disaster risk, vulnerability and resilience could significantly limit the quality and reliability of existing methodologies. Recommendations for improvements to indicator development and use are made, as well as suggested future research directions to enhance the theoretical and empirical knowledge base for composite indicator development.

## Introduction

Increasing attention is being given to issues of vulnerability, capacity and resilience in disaster management. There is a growing corpus of literature seeking to expand both the theoretical understanding of disaster resilience and vulnerability and to empirically measure it.^1^^,^ 2 ^,^ 3 ^,^ 4 ^,^ 5 One empirical approach is the use of composite indicators, which have received significant attention for their potential use as policy and communication tools.^6^^,^ 7

Composite indicators have been extensively deployed for the purpose of comparing performance of nations. The most recent comprehensive effort to catalogue these was conducted in 2008 and found 178 indices that rank or benchmark countries according to economic, political, social or environmental measures.^8^ Composite indicators have also been utilised at the sub-national level to examine a diverse range of topics including socioeconomic status,^9^ competitiveness,^10^ and sustainability.^11^

Since Briguglio published an index examining the economic vulnerabilities of small island developing states to disasters in 1995,^12^ and Cutter's 2003 publication on the development of a disaster focussed Social Vulnerability Index or SoVI 13 there has been a dramatic increase in the number of methodologies aiming to measure some aspect of disaster risk, vulnerability or resilience.

Although a number of authors have identified challenges and limitations, to date there have been only limited attempts to ascertain the current state of practice in the development of composite indicators for use in understanding disaster risk, vulnerability and resilience. A broader understanding of how composite indicators are being constructed and the variables that are being used could be of assistance to those constructing these indices by identifying common practices and gaps currently present. Policy makers could also benefit from these types of analyses; by understanding the breadth of practice they may be better able to select and critically evaluate an index that is appropriate for their needs.

A number of authors have sought to compare various disaster related composite indicators. Da Silva and Morera included seven frameworks related to disasters in their examination of 24 composite indicator frameworks relevant to different aspects of urban performance.^14^ Khazai's VuWiki has indexed about 55 methodologies but undertaken little in the way of comparative analysis, the prime focus being the development of an ontology of vulnerability frameworks.^15^ The EMBRACE project covered 32 frameworks including ecological, sociological, psychological, critical infrastructure and organisational resilience.^16^ Balica examined 10 diverse indicator methodologies with a focus on natural hazards.^17^ Schauser et al. examined 26 threat-specific indicator methodologies related to climate change.^18^ Gall compared four popular social vulnerability indices in a detailed dissertation.^19^ Birkmann examined three global and one local risk and vulnerability indicators to better understand the relevance of scale.^20^ In the earliest relevant review Pelling examined 10 tools for the measurement of urban vulnerability and risk.^21^

Despite not attempting to be a complete or even extensive survey of the literature the reviews to date have uncovered a broad range of practice in the development of community disaster risk, resilience and vulnerability composite indicators. Many have incorporated studies examining similar concepts in other domains such as psychological, critical infrastructure or organisational resilience,^16^ alternative approaches to the study of vulnerability,^15^ related concepts such as sustainability in urban areas,^14^ and the development of composite indicators for specific hazards.^18^ Some efforts, such as the VuWiki, have gone so far as to develop a detailed ontology that enables a consistent description of different studies and recent studies appear to have taken more of a cataloguing approach for the purposes of comparison rather than earlier reviews which focussed on individual methodologies in more detail. Though early reviews noted the lack of theoretical connection and justification for composite indicator methodologies,^19^ subsequent reviews have revealed more theoretical development and some well detailed frameworks notably in the grey literature.^16^

This is reflected in the very diverse conceptualisation of the components of resilience used in different indicator frameworks. One review examined a small number of frameworks associated with community disaster resilience, along with others examining resilience of socio-ecological, psychological, critical infrastructure or organisational systems. From this very diverse group the study synthesised a set of 15 components of resilience:^16^

1. Governance (Actors, Institutional Arrangements, and Organisations)
2. Education, Research, Awareness and Knowledge
3. Information and Communication
4. Culture and Diversity
5. Preparedness
6. Response
7. Protection
8. Exposure, Experience and Impact Severity
9. Resources
10. Health and Well Being/ Livelihood
11. Economic
12. Adaptive Capacity
13. Coping Capacity
14. Innovation and Capital
15. Infrastructure and Technical.

Closer examination of the concepts represented revealed that many frameworks are implicitly using transformative concepts of resilience with their focus on flexibility through learning and critical reflection. This is despite some of them seeming to focus on coping concepts of resilience with their focus on planning and preparedness. They also assessed the non-academic studies and frameworks to be more advanced in proposing frameworks to measure community resilience. Both of these factors highlight the need for care to be taken when comparing measurement frameworks, similar terminology may not have the same meaning across different studies.

One key finding from the literature in relation to the breadth of practice in the development of composite indicators is the varying motivations for the development of these tools. Three key motivations for developing indices and indicator frameworks have been identified:

- Ranking relative performance
- Influencing or driving change in performance
- Understanding and diagnosing performance

The choice, type and manipulation of the data vary for each different motivation and an approach developed for one motivation, for example to measure relative performance, is not likely to be appropriate for another motivation, for example planning and goal setting within a single city.^14^

Despite the broad variety of motivation and practice, the increasing theoretical development and the availability of guidance material for the development of composite indicators,^6^^,^ 19 there remain a number of significant challenges and limitations in the use of composite indicators for the measurement of disaster risk, vulnerability and resilience.

For example, one detailed analysis of index values found that some supposedly specialised vulnerability indices are not significantly different from broader development oriented indices such as the Human Development Index. Although some other indices are appreciably different, they are subject to significant methodological flaws such that they may not provide an adequate measure of vulnerability. Furthermore a number of indices fail to include variables, such as education and gender, which have been shown empirically and theoretically to have a strong association with vulnerability.^19^

A key challenge identified by many of the reviews in the development of composite indicators is the availability of quantitative data relevant to the conceptualisation of vulnerability that is being measured.^14^^,^ 16 ^,^ 18 ^,^ 19 ^,^ 20 ^,^ 21 The availability of data, rather than the conceptual model, may be a key or even the primary factor in the selection of variables for inclusion in an index.^20^ The availability of data is also a key issue in the development of sub-national methodologies that can be used to measure or compare the vulnerability of areas in a cross-national context.^18^ On the other hand the broad application of certain indices at multiple levels and across different contexts has been criticised as making the ecological fallacy.^19^ Some drivers, such as population density, might vary in strength and direction of influence depending on the context, making their broad inclusion in composite indicators problematic.^20 ^Although many studies have addressed the dearth of quantitative data through the use of surveys and ordinal scales,^14^ this may increase the level of subjectivity of index values and give them a perception of increased precision and accuracy. The development of truly quantitative measures of resilience and vulnerability appear to be in their infancy.^16^ By oversimplifying complex concepts such as vulnerability and resilience and using difficult to understand aggregation procedures some indicator methodologies may be reducing their utility for policy makers or even leading to poor decision making.^14^^,^ 17

The most recent review highlighted the communication of index results to policy makers as a key challenge. Consideration of the intended audience is highly important in determining if an index is appropriate and at what level of aggregation data should be presented. In the absence of agreed benchmarks it can be difficult to determine whether the index value indicates whether a government or community's performance is sufficient or needs to be improved. This can be particularly problematic when index methodologies are applied outside of the context in which they were developed. Furthermore what may be good performance in one global or national region may be poor performance in another.^14^Many studies have identified the transparency of index construction methods as an important issue both for policy makers in understanding the benefits and limitations of the index and for researchers in their critique and improvement.^14^^,^ 18 ^,^ 21 This is particularly important given that many methodologies are being published in the grey literature and thus may not be subject to the same level of scrutiny as those in the academic literature.

A key step in the index design process is the use of sensitivity and uncertainty analysis.^6^ Whilst its prevalence has not been examined in the present literature some criticism of index design has focussed on its lack of deployment and the seemingly arbitrary choices made in index construction.^19^^,^ 22 ^,^ 23 The seeming lack of both validation and sensitivity and uncertainty analysis is a problem when it comes to assessing the utility and meaningfulness of individual composite indicators and determining whether there exist any superior methodologies. In Tate's application of global sensitivity and uncertainty analysis to common index construction choices it was found that a range of these choices can lead to substantial changes in index results.^22^ This finding lends validity to those criticisms of the lack of use of sensitivity and uncertainty analysis in the development of composite indices.

Though many of these reviews have been for the purpose of improving measurement of national or community disaster resilience they have often included methodologies not related to disasters or for specific sectors or hazards. Though they have covered the breadth of practice many of the methodologies reviewed may not be generalisable into the all-hazards community resilience space. Additionally few present reviews have systematically analysed the types of data that are being used by these composite indicator methodologies, often instead focussing on the intent of their authors. Even though the intent may be to develop a disaster focussed index, the choice of variables may make the index indistinguishable from generic development and welfare indices, as found with the Predictive Indicators of Vulnerability,^19^ and thus offer very limited insight into disaster specific resilience and vulnerability. Furthermore, by examining variables related to disaster risk reduction, preparedness and resilience it may be possible to identify whether there is any agreement in the literature on what variables to examine to understand these phenomena in communities. This has the potential to feed in to the broader international discussion on measurement of progress in the Sendai Framework for Disaster Risk Reduction and the Sustainable Development Goals, which both have a substantial focus on data and measurement.

This paper seeks to expand the existing set of reviews by cataloguing the full set of disaster risk, resilience and vulnerability composite indicators and thus for the first time ascertaining the prevalence of various index construction practices and areas of implementation. Also it conducts detailed analysis of the variables included in these indices, providing new insights into what they are actually measuring.

## Methods

The wide deployment of indicators and related methodologies to study a range of phenomena related to disaster risk, vulnerability and resilience requires a strict set of criteria to enable an extensive review. It was desired to include practical methodologies that took a broad perspective on disaster risk, vulnerability and resilience. The following criteria for the review were selected:

- Composite indicator (including those on a spatial basis) or scorecard approach. These are the methodologies that are being studied – more model based approaches which produce results in terms of a 'real' number, such as average annual losses, were excluded.
- Disaster vulnerability or resilience or total risk (inclusive of social/economic aspects) is a significant focus, component or proposed application. Studies focussed on post-disaster recovery are excluded. This was chosen to ensure relevance to disasters.
- Multiple/All Hazards. This was chosen to ensure the review remained practically small as there are a large number of single hazard risk index methodologies and as single hazard risk indices tend to be much more focussed on physical risk and less inclusive of social or economic aspects.
- Communities or governments are targeted (national or sub-national in scope), not households or individuals nor single sectors.
- Full methodology is published or otherwise publicly available. This was considered important to enable analysis of the practices used in the methodology.
- Focus is on present day – climate vulnerability studies of the future are excluded.
- Framework has been tested or implemented.

An extensive search of the academic and grey literature was undertaken that used the VuWiki,^15^ Scopus, Web of Knowledge and Google Scholar, as well as forward and reverse citation searching utilising a snowball approach. A Google Web search was also conducted to capture methodologies reported in the grey literature. The search sought articles published between 1 January 1990 to 31 March 2015. The review found 265 documents of potential interest which were, upon further review against the criteria, narrowed to 126 documents detailing 106 methodologies or implementations. These are listed at Annex 1. A list of methods initially captured that were subsequently excluded is at Annex 2. Figure 1 displays the countries where the lead authors (or their institution) of these methodologies are based. A large number have been developed by researchers in the United States and Western Europe, although academics in China have been very active in index development and a small number of researchers in developing countries have also developed indices.

**Map of Authors:**
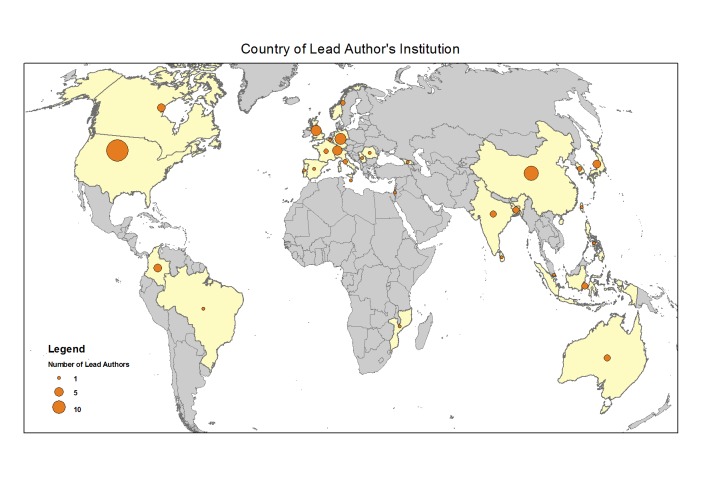
Location of lead authors or their institution for included methodologies

Data was gathered on the title, author, year of first implementation, geographic areas used, variable selection, data collection, imputation, normalisation and weighting, index structure, variables used, outputs and the use of any sensitivity or uncertainty analyses based on the review by da Silva and Morera,^14^ and the index construction practices in the OECD's Handbook on Constructing Composite Indicators: Methodology and User Guide.^6^ These were analysed to determine the frequency of different techniques implemented. The variables used in each index were recorded and grouped into sub-indicators, indicators, categories and environments based on the phenomena each variable was measuring. This classification hierarchy is illustrated in Figure 2. These were analysed to determine the frequency of use of different concepts across different methodologies as well as the composition of each methodology.

**Classification Schema:**
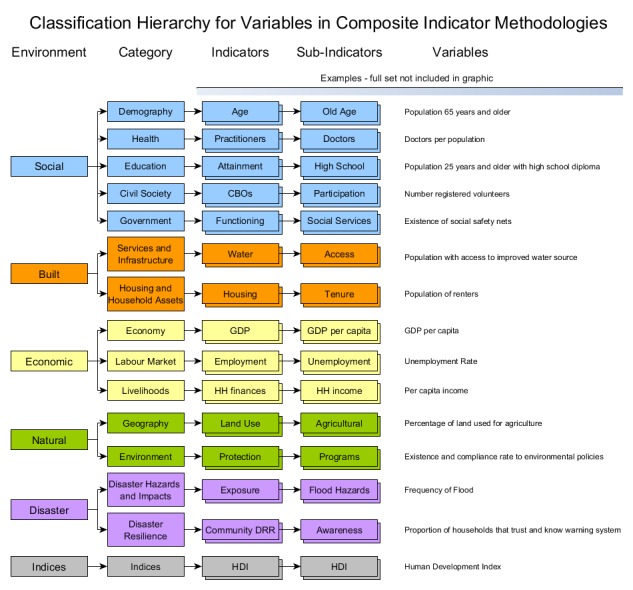
Schema used for classifying variables in the composite indicator methodologies.

## Results - Methodological Approaches

The methodologies analysed can be divided into five groups based on similar approaches to purpose, data gathering and index construction:

1. Hierarchical and Similar Deductive Methods – 70 methodologies
2. Methods using Principal Component Analysis – 17 methodologies
3. Stakeholder-focussed Methods – 10 methodologies
4. Relational Analysis Methods – 5 methodologies
5. Novel Statistical Techniques – 4 methodologies

**Hierarchical and Similar Deductive Methods**

These methods take a relatively simple deductive approach with variables combined using a hierarchical index or risk/vulnerability/resilience equation. This category contains the most variation, ranging from simple indices using a few equally weighted variables to complex, multi-layered hierarchies with weights selected through more rigorous methodologies.

**Methods using Principal Component Analysis**

Since the publication of Cutter's Social Vulnerability Index (SoVI)^13^ there have been numerous indices constructed using principal components analysis (PCA). The majority have been similar implementations of SoVI in jurisdictions outside of the USA using alternative variables, but some have used PCA in alternative contexts, including with data gathered by community survey. These methods are focussed at the sub-national level, as PCA typically requires a large number of study units to produce reliable results. As it is a data reduction technique it is also suitable for the data rich environments of developed countries where large statistical agencies collect comparable data across many small areas. PCA can be implemented in a range of different ways, with a key choice being the rotation method used in constructing the principal components. Where the rotation method used was listed they have all used varimax rotation as a means of minimising the number of factors, due a desire to attempt to explain the conceptual significance of each factor. However it is also likely that the use of a varimax rotation in Cutter's SoVI was also influential.

**Stakeholder-focussed Methods**

These methods have been mostly developed for the use of communities or governments as a self-assessment tool and as such focus on explicit elicitation of disaster preparedness and risk reduction outputs. They are typically less-focussed on index construction and in a number of cases (for example IBM/AECOM's Disaster Resilience Scorecard^24^) don't aggregate variable responses at all. They are often made entirely available to the public as some sort of toolkit, including as part of broader DRR advocacy programs (for example UNISDR's Making My City Resilient Campaign).^25^Users typically don't report on their use or their results, making it difficult to gauge the breadth of their implementation.

**Relational Analysis Methods**

These methods generate an index based on analysing the relationship between vulnerability inputs and disaster impacts using either simple or multiple linear regression or Data Envelopment Analysis (DEA).

**Novel Statistical Techniques**

Four methods in the literature used more advanced construction methods, which have not been broadly deployed. They feature novel use of statistical methods and simulation to produce the index result, which potentially makes them more difficult to understand and less transparent to end users. They are:

- The Local Disaster Index. Produced as part of the IADB suite of disaster indices the Local Disaster Index attempts to identify the impact of small-scale disasters on national and local development in a country. It utilises localised historical disaster data and a series of equations to produce an index that indicates how widespread and persistent small and moderate disasters are in a country's territory.^26^
- Geoscience Australia's Social Vulnerability. This approach developed by Geoscience Australia synthesises a vulnerability index for individual households in small areas, which is then summed to produce the area index. It uses synthetic micro-estimation with census data to produce simulated households in the area. Scenario analysis with complex decision trees, which were constructed based on population survey, was then utilised to determine individual household vulnerability. These household values were then statistically analysed to produce vulnerability values for each area.^27^
- CN-TFN. To develop a risk index in China Jin et. al. utilised triangular fuzzy numbers for weights and indicator values and Monte Carlo simulation. This method also enabled the provision of confidence estimates on the resulting vulnerability values.^28^
- Social Vulnerability Index (SVI). Ge produced a social vulnerability index using the Projection Pursuit Cluster (PPC) method, which is a data reduction and rotation methodology similar to Principal Component's Analysis.^29^

## Results - Growth in Number of Methodologies

Figure 3 shows the year of development (or submission for publication) of each methodology. In the several years after Briguglio published their index in 1995 a small number of composite indicator methodologies were published. The rate of publication increased through the middle 2000s, however during the past five years there has been a large increase in the rate of publication with close to two-thirds of composite indicator methodologies developed since 2010.

**Growth in Number of Methodologies:**
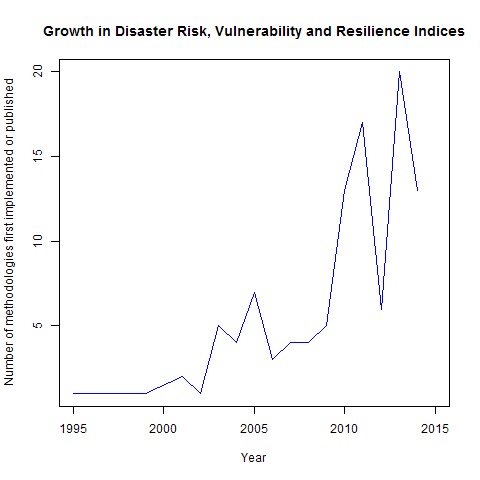
Number of new disaster risk, vulnerability and resilience indices implemented/published in each year

## Results - Geographic Level

Table 1 shows the geographic level at which each methodology has been applied. Those at the national level have studied nations or been completed on a global basis. Sub-national administrative units are studies completed in well-defined sub-national areas. Community level refers to studies in locations where the geographic boundary is not well defined or doesn't correspond to an administrative area. Multiple levels refer to studies which have applied the same methodology at both national and sub-national levels. Despite the high profile given to many indices that compare nations, they only comprise a fifth of the total. Three quarters are focussed on sub-national settings, with the majority of these based on well-defined administrative units. This is consistent with the large number of data driven methodologies, as statistical data is typically provided on the basis of these territorial units. Only a small number of methodologies have been developed for application at multiple levels. This suggests that most authors are attempting to tailor their approach to a particular level and at least in this sense are not in danger of committing the ecological fallacy, a concern that has been raised in the literature.^19^

**Table 1:** The number and proportion of methods targeted at different geographic levels

Geographic Level	Number	Percentage of total
Nation	20	19%
Sub-national administrative unit	62	58%
Community (boundary not well defined)	19	18%
Multiple levels	5	5%

## Results - Geographic Coverage

Of the 25 national or multiple level methodologies only 23 directly compare nations, with two of the multiple level methodologies taking a gridded approach to mapping the index value. Of these 23 methodologies that compared nations only eight were global in scope, with the remainder prepared for particular regions or some other sub-set of countries such as developing nations or Small Island Developing States. Seven methodologies focussed on the regions of South and Central America, West Africa, South Asia or the Asia-Pacific.

The majority of sub-national methodologies have been applied in single countries with only nine methodologies applied in a cross-national context. Only five of these are applied in more than two countries. These are the UNISDR's Local Government Self Assessment Tool (LGSAT – used in participating cities), IBM/AECOM's Disaster Resilience Scorecard (participating cities), the composite index of sub-national climate security vulnerability (Africa), the Hotspots Climate Vulnerability Index (south-east Asia) and the Integrated Vulnerability Index (Europe). The small number of methodologies that are applied across multiple countries is consistent with the literature, which has identified this approach as both difficult and a key gap in the field. There are 75 sub-national methodologies which focussed on single countries, implemented in 26 countries, with a large number in China and the USA comprising 40% of the total. These are displayed in Table 2 and Figure 4.

**Table 2:** Location of single country sub-national methods.

Country	Number of methodologies
USA	16
China	14
India	8
Indonesia	5
Germany	3
Bangladesh	3
South Korea	2
Philippines	2
Norway	2
Mozambique	2
Canada	2
Australia	2
Trinidad and Tobago	1
Taiwan	1
Sri Lanka	1
Serbia	1
Samoa	1
Romania	1
Portugal	1
Pakistan	1
Nepal	1
Japan	1
Israel	1
Georgia	1
Colombia	1
Brazil	1

**Location and number of composite indicators implemented at the sub-national level in single countries and multiple countries:**
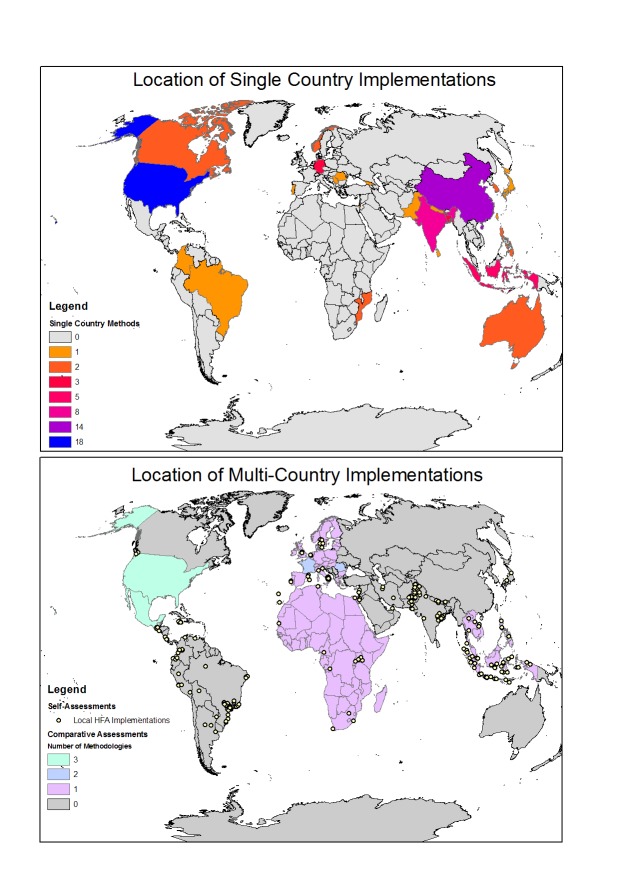

## Results - Variable Selection Methods

In the vast majority of methodologies (90) the variables were chosen by expert judgement relying on the literature, theory models and stakeholder knowledge. A smaller number (9) utilised statistical analysis (such as examining correlations to exclude redundant variables) to assist in variable selection. Three methods directly used stakeholders through workshops to select variables, whilst the remaining four used other means or a mix of approaches. In many cases developers cited variable inclusion in other indices in the literature as justification for inclusion, rather than empirical evidence or theoretical vulnerability and resilience frameworks.

## Results - Data Collection Methods

A variety of methods were used to collect data which are summarised in Table 3. The majority (76) utilised existing data collected by national statistical agencies and other government or non-government organisations that gather sociological and economic data. For national level methodologies this proportion rose to 90%, with many utilising datasets developed by the World Bank and United Nations. For sub-national methods the data in many cases came from national censuses to enable the use of small areas. Due to the use of census data which is collected relatively infrequently, the data used in a number of the indices was up to 10 years old at time of publication.

A smaller number of methodologies (10) used household surveys to gather relevant data. Although the type of data gathered by these surveys was broadly similar to those using existing statistical data they typically sought more information on disaster impacts and household/community resilience activities.

The more explicitly stakeholder focussed methodologies utilised either workshops (4) or surveys of relevant stakeholders (4) to collect information. Expert advice was relied on in only two methods. A further ten studies adopted a mixed approach to data collection utilising data from statistical agencies, expert advice and stakeholder responses.

**Table 3:** Number of methodologies using different data collection approaches

Data Collection Method	Number of Methodologies
Existing datasets	76
Community Survey	10
Stakeholder Survey	4
Workshops	4
Expert Advice	2
Mixed Approach	10

## Results - Imputation Methods

The majority of methods (96) did not perform any imputation with a small number using either case deletion (2) or some form of single imputation (8) to deal with missing data. A more important factor in dealing with missing data is that, as discussed in the literature, a number of authors acknowledged that data availability influenced variable selection.

## Results - Normalisation Methods

Many methodologies applied no normalisation to the data, either because it was not relevant to the aggregation method or because the data types were already consistent. Where normalisation was applied Min-Max methods were the most popular followed by standardisation, categorical scales and ranking. A range of other methods or mixed methods were used in other cases. The results are summarised in Table 4.

**Table 4:** Number of methodologies that use different normalisation approaches

Normalisation Method	Number of Methodologies
None	23
Min-Max methods	23
Standardisation	19
Categorical scale	17
Ranking	2
Unknown	1
Other or Mixed methods	21

## Results - Weighting Methods

A broad variety of methods for weighting variables in index construction have been deployed, including a number of bespoke methods. The results are summarised in Table 5. Equal weighting of variables and indicators appears to be the default setting in index construction, having been used by 44 index methodologies. Where equal weights were used indices were constructed to either weight each variable equally or in some multi-level hierarchies to weight each branch equally. Two of the scorecard methods did not use aggregation of the variables and don't have any weighting.

In some other cases (13) the study authors chose weights based on expert experience and the literature, but did not employ any rigorous participatory or statistical method for selecting weights. Nineteen indices used a participatory method of selecting weights. The most popular participatory method was the Analytic Hierarchy Process, used in 8 indices. Eleven studies used other participatory methods included ranking,^30^^,^ 31 DEMATEL analysis,^32^^,^ 33 ^,^ 34 the Delphi method,^35^ the Budget Allocation Process,^36^ and workshop or other interview based assignment.^37^^,^ 38 ^,^ 39 ^,^ 40

Principal Components Analysis is the most popular statistical weighting method, used by 17 methods and typically implemented using the procedure developed for Cutter's Social Vulnerability Index. In most cases (10) PCA factors were aggregated with equal factor weights. Three methods used the factor score (which indicates the percent of variance explained) as the factor weightings, three did not aggregate the factors and one was unclear. Other statistical methods employed include triangular fuzzy numbers and stochastic simulation,^28^ data envelopment analysis,^28^^,^ 41 ^,^ 42 multivariate regression against other indices,^28^^,^ 43 multivariate regression against outcome measures,^44^^,^ 45 ^,^ 46 microsimulation and decision tree analysis,^27^ the projection pursuit cluster model,^29^ the procedure developed for the Disaster Deficit Index,^47^ and the procedure developed for the Local Disaster Index.^48^

**Table 5:** Use of different weighting approaches by composite indicator methodologies

Weighting Method	Number of Methodologies
Equal weights	44
Selected by authors	13
Selected by workshop/interview	4
Analytic Hierarchy Process	8
Ranking	2
Delphi Method	1
DEMATEL analysis	3
Budget Allocation Process	1
Principal Components Analysis – equally weighted factors	10
Principal Components Analysis – weighted by factor scores	3
Principal Components Analysis – no aggregation	3
Principal Components Analysis – unknown	1
Data envelopment analysis	2
Multi-variable regression	4
Project pursuit cluster model	1
Bespoke statistical methods	4

## Results - Index Construction and Aggregation

A variety of inductive and deductive approaches were used to construct the indices. Three approaches used no aggregation, three of the principal components approaches just provided the factors and used no further aggregation, 74 used a hierarchical approach, 14 combined factors from principal components analysis, whilst the remainder employed other mostly relational approaches.

Of the hierarchical indices the majority (59) used a simple structure with aggregation at each level by arithmetic or geometric (weighted) mean. In the remaining 15, the theoretical models incorporated a risk, vulnerability or adaptive capacity equation which was typically at the top of the hierarchy, the equation's components being constructed as the weighted mean of the indicator variables. Some of the methodologies based on PCA also employed a hierarchical approach in the combination of the factors, once they had been calculated. The hierarchical models typically used a single intermediate level between variables and index in the hierarchy (median=mode=1), however the methods that mixed simple hierarchy with an equation were more complex, typically with 2 (median=mode=2) intermediate levels. The most complex model is the Index for Risk Management (InfoRM) which included five intermediate levels.^49^ The greater the number of levels in the hierarchy the greater the number of variables included, although this varied substantially as shown in Table 6.

**Table 6:** Numbers of variables in deductive methodologies with different numbers of intermediate levels between the variables and the reported index

Number of levels	Number of Methods	Smallest number of variables	Median number of variables	Largest number of variables
0	20	2	7	56
1	28	4	24.5	55
2	19	8	24	125
3	5	20	55	81
4	1	-	52	-
5	1	-	54	-

## Results - Presentation of Outputs

Almost all methodologies provided some display of the results with maps and tables being the most popular as summarised in Table 7. Only four methodologies provide an interactive display of the results, the Index for Risk Management (InFORM), Economic Vulnerability Index, the Rural Resilience Index and the Resilience Capacity Index. A small number of methods published no output whatsoever.

**Table 7:** Number of methods presenting outputs in different forms

Output Format	Number of methods using format
Tables or Summary Statistics	55
Charts	33
Maps	66
No Results	7
Interactive Output	4

## Results - Sensitivity and Uncertainty Analysis

Although many papers discussed the potential limitations of the methodology developed, only twenty have any explicit analysis of uncertainty or sensitivity. The most commonly employed sensitivity analysis was the application of different weights to the indicators/variables or use of a different weighting method with nine methodologies taking this approach. Three methodologies explored the sensitivity to alternative aggregation methods. Two assessed the use of different groups of variables. Only two methodologies provided estimated errors on the resultant vulnerability scores. Of the methodologies included in this study only one comprehensive sensitivity analysis was undertaken, for Cutter's SoVI^50^ which incorporated an investigation of different geographic levels and 54 unique variations on the index construction. However other work that failed to meet the inclusion criteria has also examined global approaches to sensitivity and uncertainty analysis.^22^^,^ 23 Five composite indicator methodologies underwent some level of validation, in three cases against disaster output information. One other compared the results against ethnographic assessment and one community survey methodology validated the resulting index against a broader set of survey questions. There was some variation in the findings of the sensitivity analyses that were undertaken, with some finding low sensitivity to changes to the methodology and others finding high sensitivity to change.

## Results - Number of Variables

There was a large variation in the number of variables each methodology used with the minimum being 2 and the maximum being 235, however most methodologies used relatively few with two thirds using less than 40. The distribution of variables is illustrated in Figure 5.

**Number of Variables:**
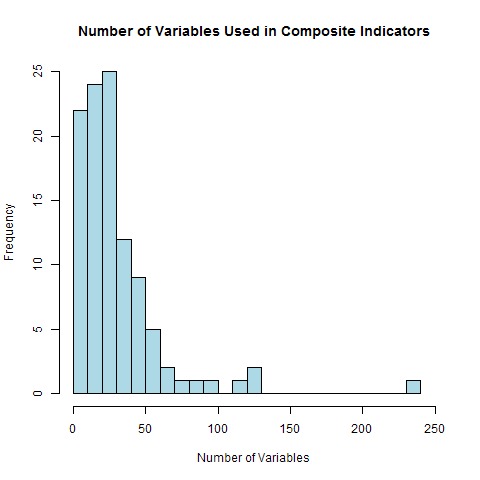
Frequency of use of different numbers of variables in composite indicators

Most of the methodologies employed mining of national and international statistical databases. Methodologies using this data collection technique used the fewest variables on average (median 18, minimum 2, maximum 68). Some of these techniques included a stage of variable exclusion based on correlation (or other statistical) analysis. Those using community surveys collected more variables (median 28.5, minimum 21, maximum 81) with those relying on expert advice (median 30, minimum 24, maximum 36) and mixed methods (median 27, minimum 8, maximum 92) collecting a similar number. However the stakeholder focussed methods (using surveys or workshops) relied on substantially more variables (median 87.5, minimum 21, maximum 235) than any other data collection technique.

## Results - Prevalence of Variables, Sub-Indicators, Indicators and Categories

The 106 methodologies used 3209 variables of which 2298 were unique. An analysis of their frequency of occurrence found that the most used variables were dominated by common statistical indicators. This is consistent with the dominant data collection methodology. The 11 most common variables are shown in Table 8.

**Table 8:** Most commonly used variables across all the methodologies

Variable Name	Number of methodologies
Population Density	33
Unemployment Rate	31
Population 65 Years and Older	19
GDP per Capita	19
Percent Female Population	18
Doctors per Population	16
Literacy Rate	15
Total Population	14
Beds in Hospitals per Population	14
Percent Individuals Below Poverty Line	12
GINI Index	12

As per the classification hierarchy shown in Figure 2, the variables were grouped under 334 Sub-Indicators, an average of 6.9 variables per Sub-Indicator. The most common Sub-Indicators were strongly influenced by the most common variables with some Sub-Indicators, such as Household Water Access appearing despite not having component variables in the top 11. This appears to be due to the larger number of ways of measuring certain sub-indicators. The 10 most common sub-indicators are shown in Table 9.

**Table 9:** Most commonly used sub-indicators across all the methodologies

Sub-Indicator	Number of methodologies
Population Density	38
Unemployment	37
Old Age	30
Population in Poverty	27
Household Water Access	27
Literacy Rates	26
Young Age	24
Typical Household Income	24
Housing Tenure	23
Warning Systems	22

The Sub-Indicators were grouped under 104 Indicators, an average of 3.2 Sub-Indicators and 22 variables per Indicator. At the Indicator level in the classification hierarchy the significant use of various variables describing age distribution in communities and properties of housing stock was demonstrated. The 10 most common indicators are shown in Table 10.

**Table 10:** Most commonly used indicators across all the methodologies

Indicator	Number of methodologies
Age	54
Housing	44
Household Finances	43
Water	42
Employment	41
Population Density	38
Hazards and Exposure	38
Transport	37
Poverty	35
Disaster Impacts	34

The Indicators were grouped under 15 categories. The number of methodologies that included variables from each of the categories is shown in Table 11. This demonstrates that a majority of the methodologies included some measure of demographics, education and health, with existing indices and measurement of aspects of government and the environment being used the least.

**Table 11:** Number of methodologies using variables in each of the 15 categories

Category	Number of methodologies
Demography	87
Education	67
Health	64
Services and Infrastructure	61
Economy	59
Disaster Hazards and Impacts	59
Labour Market	47
Livelihoods	47
Housing and Household Assets	47
Disaster Resilience	41
Civil Society	39
Geography	37
Environment	28
Government	24
Indices	21

Looking at the number of Indicators, Sub-indicators and Variables in each category and the proportion of variables that are used more than once it is possible to better understand how much variety within the literature there is in terms variables to represent these different concepts. The proportion of variables used more than once is indicative of the level of agreement in the literature on what variables to measure to understand the properties of that category. This is shown in Table 12. In most categories, at most one-third of the variables were used more than once, however this falls to 8% for measuring aspects of government and 5% for measuring aspects of disaster resilience. Not only was disaster resilience the category with the fewest variables used more than once, but it also had the largest number of unique variables, representing 21% of all variables.

**Table 12:** Number of Indicators, Sub-Indicators and Variables in each category and the proportion of variables in each category that are used in more than one methodology

Category	Indicators	Sub-Indicators	Variables	Proportion of variables used more than once
Demography	17	50	186	32%
Education	8	22	78	23%
Health	17	38	131	24%
Services and Infrastructure	12	36	282	20%
Economy	7	24	121	22%
Disaster Hazards and Impacts	3	25	286	20%
Labour Market	5	15	80	20%
Livelihoods	2	13	122	20%
Housing and Household Assets	2	15	89	31%
Disaster Resilience	8	32	492	5%
Civil Society	4	14	140	11%
Geography	3	13	59	36%
Environment	2	8	97	10%
Government	3	11	77	8%
Indices	11	18	58	24%

The 15 categories were grouped into 6 environments, to better enable visual analysis of the composition of each index. The use of variables in these 6 different environments in the different methodologies is summarised in Table 13.

**Table 13:** Number of methodologies containing variables in each environment

Environment	Number of methodologies
Social Environment	98
Built Environment	74
Economic Environment	86
Natural Environment	51
Disaster Environment	75
Indices	21

The most common variables are related to various social aspects of communities especially demographics, education and health. Respectively population density, number of doctors and literacy rate were the three most common variables in these categories. Variables representing various economic aspects of communities: livelihoods, labour market and economy were the next most common. Respectively per capita income/per capita welfare receipts, unemployment rate and per capita GDP were the most common variables in these categories. Variables measuring housing, household assets, services and infrastructure were also very common, present in 70% of the methodologies. The number of renters and access to clean water were, respectively, the most common variables in these two categories. Despite purporting to measure disaster risk, vulnerability or resilience only 75 (71%) of the methodologies included some measure of disaster hazard, impact or resilience. Existing indices were used in only 21 of the methodologies, with most relying instead on directly collected data. However despite the risk of double-weighting, by also including variables that are already present in an included index this appears to have only occurred in two methodologies. One included both GDP per capita and the Human Development Index,^51^ and another included the Multidimensional Poverty Index and some of its constituents.^49^

## Results - Composition of Indices

Although the prevalence of different variables provides some insight into their popularity in disaster risk, vulnerability and resilience indices it does not reveal the make-up of the individual indices. To better understand their composition the proportion of the variables classified into each environment was calculated for each index. The results of this classification are displayed in Figure 6 and the averages across all the indices displayed in Table 14. These show that most indices are dominated by variables related to the social environment, with a much smaller number using high proportions of variables from the disaster environment. An average of 25% of variables in all the indices (and 35% in those indices that used them) were related to disasters. However when disaster resilience variables are specifically examined (i.e. only those variables measuring action to mitigate or prepare for disasters) this falls to 12% of all indices on average and 30% of those indices that included these direct measures of disaster resilience.

**Composition of Indices:**
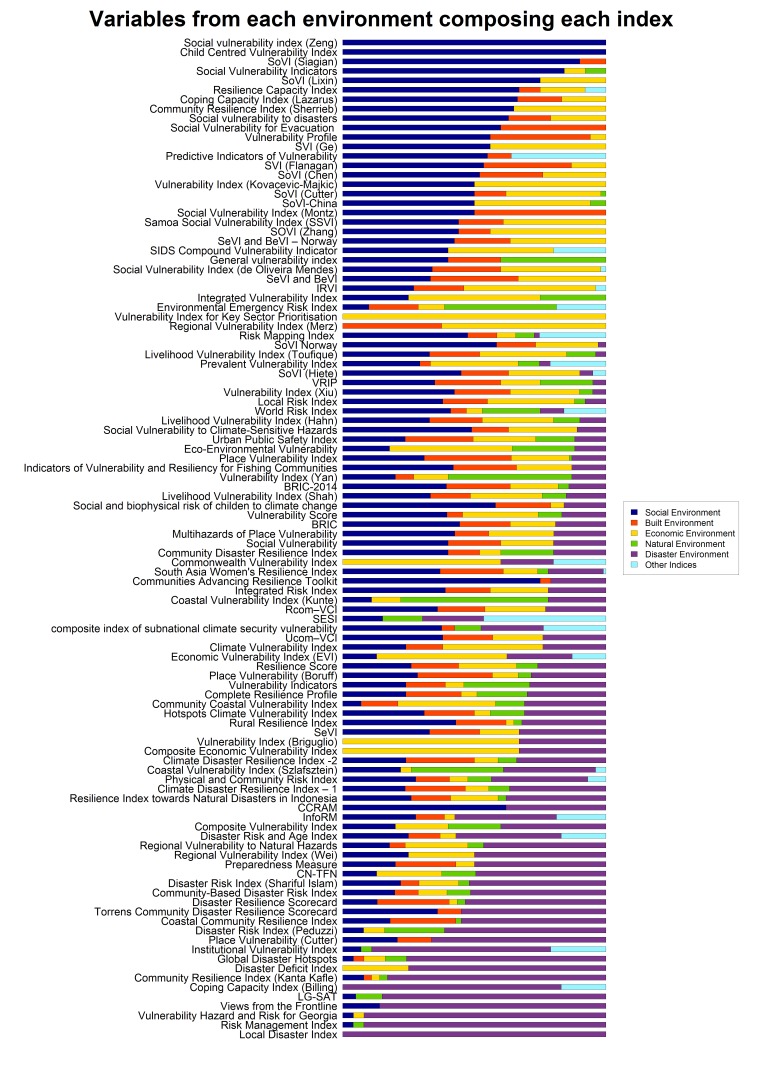
Variables that compose each index classified into one of six environments as a proportion of the number of variables in each index.

**Table 14:** Proportion of variables from each environment that comprise each index, on average, for all methodologies and for methodologies that only include variables in that environment

Environment	Average proportion of variables in each index	Average proportion of variables in each index for indices with variables from that environment
Social Environment	34%	36%
Built Environment	13%	18%
Economic Environment	20%	24%
Natural Environment	6%	13%
Disaster Environment	25%	35%
Indices	3%	16%

Examining the correlations between the proportions of variables included (Table 15) shows the strongest relationship between the social and disaster environments; the more social variables that are included, the fewer disaster variables that are included.

**Table 15:** Correlations between proportions of variables in each environment (correlations that are significant at p<0.05 for Pearson's r ns=not significant)

Correlation	Social	Built	Economic	Natural	Indices
Built	ns				
Economic	-0.19	ns			
Natural	-0.26	ns	ns		
Indices	ns	-0.23	ns	ns	
Disaster	-0.63	-0.34	-0.40	ns	ns

It is also possible to compare how the proportion of variables in each index varies according to the type of methodology which is shown in Table 16. Only the Deductive, PCA and Stakeholder-based methodologies have been included due to the small number of methodologies in the Relational and Novel techniques categories. This demonstrates that there is some variation in the type of variables used in these methodologies depending on the approach used. Although Deductive and PCA approaches appear to be broadly similar in the proportions of variables included, PCA approaches feature far fewer disaster related variables. Stakeholder based approaches include many more disaster related variables, making up approximately half of the variables in these methods with a much lower focus on economic variables.

**Table 16:** Proportion of variables from each environment present in methods using the three most common construction approaches

Environment	Deductive	PCA	Stakeholder
Social Environment	33%	49%	27%
Built Environment	12%	19%	14%
Economic Environment	21%	23%	4%
Natural Environment	8%	1%	4%
Disaster Environment	22%	7%	51%
Other Indices	5%	0%	0%

## Results - Commonality between the variable sets chosen in each methodology

It is desirable to know whether the large number of composite indicator methodologies is actually adding new explanatory power to understanding of vulnerability, risk or resilience or whether they are repeatedly using the similar sets of variables and only varying the construction method. Ideally this would be tested by comparing index values for the methodologies in the same area, but with little geographic overlap and data unavailability this would not be practical. To gauge the amount of variation in the choice of variables across all the methodologies a custom measure, the 'Overlapping Score' was created to measure the proportion of elements in common at each level of the classification hierarchy (variable, sub-indicator, indicator, category, environment).

\begin{equation*}X=\frac{1}{n^2} \sum_{i=1}^{n}{\left( \sum_{j=1}^{n}{\frac{\sum_{k=1}^{m}{min(x_{ik},x_{jk}) } }{min\left( \sum_{k=1}^{m}{x_{ik}},\sum_{k=1}^{m}{x_{jk}} \right)  } }-1  \right) } \end{equation*}

where *n* is the number of methodologies, *m* is the number of variable, sub-indicator, indicator, category, environment (or indicator group), *x_ik_* is the number of indicators for the *k*th indicator group in the *i*th methodology and the results are given at Table 17. This index has been constructed such that a set of methods using identical variables, sub-indicators etc. would give a score of 1 and a set of methods using entirely unique variables, sub-indicators etc. would give a score of 0.

**Table 17:** Overlapping score calculated for each level in the classification hierarchy

Level	Overlapping Score (X)
Variable	0.04
Sub-Indicator	0.12
Indicator	0.24
Category	0.50
Environment	0.71

These results show that there is relatively low internal consistency between the methodologies when measured at the variable level this increases substantially when considering the indicator level and above. This suggests that many of these methodologies may not offer substantially different results in presenting an understanding of risk, vulnerability or resilience. By examining the average of the absolute values of the correlation of each method against all others it is possible to uncover those methods that include a more unique set of variables – this was applied at the indicator level. The four most unique methodologies are:

- Predictive Indicators of Vulnerability^52^
- Communities Advancing Resilience Toolkit^53^
- Local Disaster Index^26^
- Disaster Deficit Index^47^

These methodologies had fewer indicators in common with the rest of the set, partly due to a focus on more unique concepts and partly because they use a relatively small number of variables. Similarly the least unique methodologies with the highest proportion of elements in common were the two versions of Joerin's Climate Disaster Resilience Index.^30^^,^ 54

## Discussion

This review has revealed a broad range of practice in the development of composite indicators for the measurement of disaster risk, vulnerability and resilience. There is substantial diversity in the literature, with a range of variable selection approaches, data collection methods, normalisation methods, weighting methods, aggregation approaches and variables being used. However this review has also identified a number of trends which may limit the utility of composite indices in improving the understanding of these concepts.

**The Broad Methodology Types**

Although the review found considerable diversity in the methodologies of index construction the majority take a fairly standard deductive or hierarchical approach with a weighted sum of the variables included in the index. In most cases the main point of difference in index construction was the choice of variables for inclusion. Hierarchical approaches are easy to construct and are relatively simple to understand which may largely explain their prevalence. Principal Components Analysis was also commonly employed, with many cases being strongly influenced by the 2003 publication of Cutter's Social Vulnerability Index (SoVI). In a number of instances its use has extended beyond addressing some of the problems associated with collinearity in deductive indices to more detailed analysis of the principal components and their spatial variation, thus taking advantage of PCA as a data reduction tool. PCA has only been applied at a sub-national level, however it is likely that comparing nations would not offer significant advantages over other methods as for it to be statistically valid only a small number of variables could be included. Stakeholder based methods were less popular than the deductive methods or PCA, however appeared to be more targeted with more variables directly related to disaster resilience. The large number of variables gathered in the stakeholder focussed methodologies could be problematic. Although a number of these are checklist-based self assessments, the volume of questions could lead to little attention being paid to responses and overall disengagement from the process. The literature generally agrees that resilience is not a 'check the box' approach but is related to systemic performance,^55^ which these methods may not focus on. Because many stakeholder based approaches are self assessment and others are being driven by a single small research group it is difficult to ascertain their full geographical coverage. Aside from pilot locations, their implementation is often not reported. This makes it difficult to assess implementation difficulties or conduct reliability analysis to identify a shorter list of questions. Relational techniques, such as linear regression and data envelopment analysis have received some attention for both the comparison of nations and sub-national areas. Although the indices that use relational methods are internally validated they have a number of potential problems that could limit their application. Data on disaster impacts may be of poor quality and have limited time coverage to adequately reveal the relationship with variables. Indices produced with these methods should be accompanied by robust uncertainty estimates that incorporate both aleatoric and epistemic uncertainty to better enable assessment of their reliability. A small number of composite indices have taken more advanced approaches, some of which are closer to more model based assessments of disaster risk. Greater sophistication in this area may produce indices that better reflect theory models. However these 'black box' methods may be more difficult to understand for end-users and promote a less critical acceptance of the results.

**Problems with Repeatability**

The review of the literature found 24 methodologies that were not sufficiently well described to include in the analysis. Although these included a number of proprietary rankings, many methods published in the academic literature also failed to provide sufficient detail to enable analysis, let alone replication. Even methods included in the review failed to include sufficient detail that would enable reconstruction of the index. Where information was sought from statistical databases, some methodologies are unclear about the agency from which the data was sourced and the years to which variables refer to. Some studies did note the year from which the data was sourced, which was up to 10 years prior to publication potentially making the index out-of-date. This has implications for decision makers using the index results. Although demographic variables may change relatively slowly this lack of currency was often not well communicated in the results of these studies, nor were attempts made to 'nowcast' the index values. Those based on community or stakeholder survey often did not include the questions asked, making comparison with other surveys difficult.

**Sensitivity and Uncertainty Analysis**

Numerous researchers have been pointing out flaws in index construction and calling for greater use of sensitivity and uncertainty analysis for quite some time as outlined in the introduction. This study has found that these calls have been largely unheeded. Despite the large increase in number of disaster risk, resilience and vulnerability composite indicators being developed, there has not been an increase in the use of sensitivity and uncertainty analysis. Few methodologies are undertaking explicit sensitivity and uncertainty analyses and where it is undertaken it is typically limited to one or two aspects of the methodology. This makes it difficult to assess quality, especially when many choices in index construction appear to have been made arbitrarily or with limited justification. Some sensitivity analyses have found significant impact of methodological choices on the resulting index values which has implications on the broader use of these indices by policy-makers. Without appropriate caveats or the provision of uncertainty estimates, decision makers may believe the index results to be much more reliable than they actually are.

**Outputs**

Most studies communicated results for example by using maps and summary tables. However many did not provide full numerical results. Although full reporting on index results for large numbers of study units is difficult in the academic literature more effort needs to be made to make these available to enable improved review of these studies, for example by comparing different index results for the same set of territories. Furthermore only 4 methodologies provide interactive portals to access and visualise the data in graphical, map or table form. Interactive options may be more preferred by policy makers, the community and others interested in the results of these studies which suggests that many authors may not be making them useful for end-users.

**Direct Measurement of Risk, Resilience and Vulnerability**

This study aimed to review composite indices that claim to measure disaster risk, vulnerability and resilience. However the inclusion of variables directly related to disasters is quite limited with 29% not including any. While 56% of the methods incorporated measures of hazards and impacts only 39% included direct measures of disaster risk reduction and preparedness activity. Where disaster related variables were included they did make up a moderate proportion (35%) of the total number of variables in the index but this dropped when considering variables related to risk reduction and preparedness. The limited use of disaster related variables is likely to be due to limited availability. This is supported by the fact that those methodologies that directly elicited information from stakeholders included substantially more of these variables than those that depended more on data gathered from statistical databases. This suggests that some of these indices may not be producing results that are qualitatively different from more common indices of social and economic advantage/disadvantage. However without access to the results of all the indices in this review and a social disadvantage index for comparison, this is difficult to assess.

Analysis of the inclusion of the variables found a strong negative correlation between use of social variables and use of disaster variables. This suggests that social variables are the proxy of choice when access to more disaster related data is difficult, with demographic variables being very common.

The limited agreement in how to measure aspects of disaster prevention and preparedness (only 5% of these variables were used in more than one methodology) may be a barrier to the greater use of this type of data. A large number of these variables are collected through workshops and stakeholder and community surveys. Alternative formulations of questions on disaster resilience, for example customised to the terminology and context of a particular jurisdiction, may limit the broader applicability of these methodologies. Work surrounding the implementation of the post-2015 development agenda, such as the development of a new "10 Essentials" may assist in addressing this gap.

**Comparison of Sub-National Areas Across Nations.**

Whilst many methods exist to compare the risk/vulnerability/resilience of nations and sub-national areas, few have attempted to compare sub-national areas across different nations. This highlights the difficulty in adapting tools across national contexts, particularly where those tools utilise data from statistical agencies which may not be available for similar scales, forms and time periods across multiple nations. The most broadly applied sub-national tool is the UNISDR's Local Government Self-Assessment Tool, which utilises fairly broad questions. An updated tool, consistent with the new 10 Essentials may be beneficial in improving cross-national coverage although the differing contexts of nations may prove too difficult for this tool to enable meaningful comparison.

**The Tension between Comparison and Self-Assessment**

Two key motivations have emerged from this analysis of composite index and dashboard methodologies of disaster risk, vulnerability and resilience. Many methods use primarily statistical data to compare large numbers of study units. Consistent comparison of different jurisdictions may be desired by national and international organisations seeking to inform decisions on resource allocation. Another group of methods seek to provide a tool, primarily to sub-national authorities, for self-assessment of disaster vulnerability through the asking of targeted questions. Self-assessment may be desired by national and local governments and communities seeking to improve their performance.

These two groupings are consistent with the three key motivations found by da Silva and Morera of ranking relative performance and diagnosing performance and influencing change.^14^ Consistent with their findings, these two groups do not appear to be compatible with one and other, especially as the self-assessments tend to be qualitative versus the quantitative approach taken by comparative assessments. There does not appear to have been any efforts to make the results of self-assessments more comparable. This could be achieved by establishing quantitative measures of performance where each assessing authority would choose their own benchmarks. However this may not produce desirable results; the use of self-assessments for resource allocation could potentially bias them towards assessing their performance as lower than reality to enable greater access to resources.

**Lessons for End-Users**

The number and variety of composite indicator methodologies that have been developed clearly indicate their potential end use for decision makers working in disaster risk reduction, humanitarian and emergency response, civil protection or other fields related to disaster resilience. However the limitations of the present literature have a number of implications for end users and there is a risk that biases and uncertainty may lead to inappropriate decisions. To counter this risk end users should consider multiple techniques when attempting to understand community vulnerability and resilience. To gain the broadest understanding this should include qualitative and quantitative techniques that go beyond composite indicators, for example using tools that are part of the Vulnerability and Capacity Assessment process developed by the International Federation of Red Cross and Red Crescent Societies. End users can also take steps to ensure that composite indicator frameworks they are using are of high quality and reliability when they are selecting an existing index for use or commissioning the development of one either internally or by an external team of experts. Composite indicator frameworks with high quality and reliability are likely to have:

1. Consideration of the purpose of the composite indicator framework, in particular whether it is needed for comparison of many areas or for local self-assessment.
2. Demonstration that the disaster specific index adds value to the discussion of risk, vulnerability or resilience. This may come from the inclusion of multiple variables that directly relate to the phenomenon of interest or a comparison of the index with a generic socio-economic status index.
3. Full publication of the methodology and results. Interested third parties should be able to replicate, evaluate and build-upon the results of composite indicators. Particular attention should have been paid to clearly specifying data sources, including agency, year and the wording of any survey questions used. This may be particularly important for increasing transparency in government decision making.
4. The results in a range of formats. Results should be published as tables, graphs and maps to enhance understanding and available in downloadable machine readable formats. Interactive displays and dashboards may also be highly useful to end users.
5. Adequate sensitivity and uncertainty analysis. This should incorporate, as far as possible, global analysis of sensitivity to understand which construction choices contribute most to possible variance in index values and uncertainty estimates for all index values.
6. Attempts to validate the index values. Although relational indices are internally validated, efforts should be made to relate other indices to outputs or outcomes relevant to the phenomena of concern. This may include disaster impacts or surveys of experts or community members on their opinion of overall community disaster risk, vulnerability or resilience.

## Conclusion

An extensive review of disaster risk, vulnerability and resilience composite indicator methodologies has been conducted drawing on a range of sources in both the academic and grey literature. The review has revealed a broad diversity of practice with implementations at both the global and local level and within many different countries. The significant increase in the number of methodologies being implemented over recent years demonstrates greater availability of composite indicators for use by researchers and policy makers. However present practice has two key limitations that may restrict their use or potentially lead to poor decisions being made in their implementation - low use of direct measures of disaster resilience and low use of sensitivity and uncertainty analysis.

Very few studies are implementing comprehensive sensitivity and uncertainty analysis, nor communicating it to end users. This may lead policy-makers to believe that index results are more precise and accurate than is actually the case. Were a comparative index to be used by a government to allocate resources for disaster risk reduction, without consideration of its reliability, it could lead to waste of government resources or possibly even increased risks if existing resources are shifted away from high risk areas. The lack of sensitivity and uncertainty analysis may be compounded by the low use of variables directly related to disaster risk reduction, preparedness and resilience. This low use of more direct variables may limit the explanatory power of these tools. Indices lacking direct measures of disaster resilience may be indistinguishable from more general measures of socioeconomic status, such as the Human Development Index, and thus may not offer increased value to researchers investigating disaster vulnerability. Lack of sensitivity analysis means that the exclusion of disaster related variables may go unquestioned by policy makers or researchers using such an index, increasing the risk of inappropriate use. Policy makers and others who wish to use composite indices to inform decision making need to critically evaluate their quality and reliability before their use. Consideration of the features of high quality and reliable indices, as outlined in the discussion, would assist decision makers to commission or select an appropriate index for their needs. Similarly, researchers developing these indices need to make greater efforts to ensure that they are relevant to the needs of decision makers, are of high quality, and add value to the understanding of vulnerability and resilience. In particular they should demonstrate that their index has greater explanatory power of disaster risk, vulnerability or resilience than generic socioeconomic status indicators and incorporate robust sensitivity and uncertainty analysis.

Furthermore, the low use of direct measures of disaster resilience may be related to the limited agreement between the methodologies of which direct measures to use. This limited agreement appears to reflect a broader gap in disaster research on the drivers of disaster resilience. It is unclear which variables, in which situations, matter most to disaster resilience. Current approaches appear to be largely tailored to individual contexts and broadly incompatible with one and other. This could be a significant barrier for achievement of the Sendai Framework for Disaster Risk Reduction, disaster related targets of the Sustainable Development Goals and other elements of the post-2015 development agenda as parties seek to agree indicators to measure performance towards targets in these agreements. Further research is needed to better identify which variables are most predictive of disaster risk, vulnerability and resilience and in which contexts they apply. This would enable the construction of more relevant and targeted composite indicators, which combined with improvements in practices related to their construction would lead to indices that are robust, fit for purpose and comparable improving the understanding of disaster risk, vulnerability and resilience and providing decision makers with tools to better monitor progress towards a disaster resilient society.

## Competing Interest

The author has declared that no competing interests exist.

## Annex 1 - List of Methods Analysed

Link to external file

## Annex 2 - Excluded Methods

Link to external file
