# Correction: A Comparative Analysis of Disaster Risk, Vulnerability and Resilience Composite Indicators

**DOI:** 10.1371/currents.dis.19f9c194f3e3724d9ffa285b157c6ee3

**Published:** 2017-06-29

**Authors:** PLOS Currents

## Correction

The links for Annex 1 and Annex 2 are incorrect. The correct links are provided here.

## Annex 1 - LIST OF METHODS ANALYSED

Link to external file

## Annex 2 - EXCLUDED METHODS

Link to external file
